# Efficacy and Safety of Antidiabetic Agents for Major Depressive Disorder and Bipolar Depression: A Meta-Analysis of Randomized, Double-Blind, Placebo-Controlled Trials

**DOI:** 10.3390/jcm13041172

**Published:** 2024-02-19

**Authors:** Jian Zhang, Rongyi Sun, Yang Cai, Bo Peng, Xi Yang, Keming Gao

**Affiliations:** 1 Shenzhen Mental Health Center, Shenzhen Kangning Hospital, Shenzhen 518020, China; zhjjjjjj@126.com (J.Z.);; 2 Mood Disorders Program, Department of Psychiatry, University Hospitals Cleveland Medical Center, 10524 Euclid Ave, 12th Floor, Cleveland, OH 44106, USA; 3 Department of Psychiatry, School of Medicine, Case Western Reserve University, Cleveland, OH 44106, USA

**Keywords:** bipolar disorder, depression, antidiabetic, meta-analysis

## Abstract

Background: This meta-analysis aimed to determine the efficacy and safety of antidiabetic agents in the treatment of major depressive disorder and bipolar depression. Methods: Randomized controlled trials (RCTs) of antidiabetic agents in major depressive disorder or bipolar depression were searched in three electronic databases and three clinical trial registry websites from their inception up to October 2023. The differences in changes in the depression rating scale scores from baseline to endpoint or pre-defined sessions, response rate, remission rate, rate of side effects and dropout rate between antidiabetic agents and placebo were meta-analyzed. Results: Six RCTs involving 399 participants were included in the final meta-analysis, which did not find that antidiabetics outperformed the placebo in reducing depressive symptoms. The standardized mean difference (SMD) in the depression scores from baseline to endpoint was 0.25 (95% CI −0.1, 0.61). However, a subgroup analysis found a significant difference between antidiabetics and placebos in reducing depressive symptoms in Middle Eastern populations, with an SMD of 0.89 (95% CI 0.44, 1.34). Conclusions: The current meta-analysis does not support the efficacy of antidiabetics being superior to the placebo in the treatment of unipolar and bipolar depression. However, a subgroup analysis indicates that patients from the Middle East may benefit from adding an antidiabetic medication to their ongoing medication(s) for their depression. Larger studies with good-quality study designs are warranted.

## 1. Introduction

Mood disorders are highly prevalent worldwide, with an immense economic burden [1,2]. Although various strategies have been developed to improve treatment outcomes in the last several decades, a significant number of patients with a mood disorder, especially during the depressive phase of bipolar disorder (bipolar depression) or major depressive disorder (MDD), do not fully respond to the currently available therapies. Current treatments of MDD and bipolar depression mainly focus on the regulation of neurotransmitters (antidepressants, mood stabilizers, antipsychotics, and ketamine) and brain circuitries (electroconvulsive therapy, repetitive transcranial magnetic stimulation, and deep brain stimulation). Previous efficacy and effectiveness studies have demonstrated the limitations of antidepressants [3,4,5,6,7,8,9,10], mood stabilizers [11], antipsychotics [12,13], ketamine infusion [14], and nasal esketamine [15,16,17] in the treatment of MDD. According to STAR*D (Sequenced Treatment Alternative to Relieve Depression) reports, only 67% or even less of depressed patients remitted after various therapies, including tricyclic and monoamine oxidase inhibitor antidepressants [18,19]. Similarly, medications for bipolar depression not only have limited efficacy but also short- and long-term safety concerns [11,20]. Among the current approved medications for bipolar depression, some showed robust efficacy but with troublesome safety concerns [21]; some showed modest antidepressant activity [22], and others had limited evidence [23,24]. As a lesser invasive interventional treatment, the efficacy of repetitive transcranial magnetic stimulation (rTMS) in unipolar or bipolar depression is not robust [25,26,27,28,29,30]. Electroconvulsive therapy, vagal nerve stimulation, and deep brain stimulation are more invasive procedures and commonly reserved for patients who are in the late stage of treatment-resistant depression [31]. In addition, cognitive dysfunction is the most common residual symptom in mood disorders [32,33,34]. However, no available treatments have demonstrated efficacy in reducing cognitive impairment in either bipolar disorder or MDD [35,36,37].

The limitations of currently used treatments have propelled researchers to study and discover new medications for depression with different mechanisms and to investigate drug repurposing for the treatment of mood disorders. In the drug repurposing for the treatment of MDD, a number of drugs from different classes have been investigated, with some drugs showing efficacy in reducing depressive symptoms [38]. The drugs showing antidepressant efficacy include ketamine (an anesthetic), pramipexole (dopaminergic agonist for Parkinson’s disease), oxytocin, doxycycline (an antibiotic), and pioglitazone/rosiglitazone (antidiabetics). In the drug repurposing for the treatment of bipolar disorder, allopurinol (lowering uric acid), melatonin, ramelteon (a hypnotic), and tamoxifen (a selective estrogen receptor modulator for treating breast cancer) were studied for mania and acetylsalicylic acid (an anti-inflammatory drug), celecoxib (an anti-inflammatory drug), N-acetylcysteine (a plant antioxidant), pioglitazone, memantine (an NMDA receptor antagonist for treating dementia), modafinil/armodafinil (non-amphetamine stimulants for treating excessive sleepiness), pramipexole, and inositol (a supplement) were studied in bipolar depression [39]. Among the drugs studied for mania, the efficacy of allopurinol and tamoxifen was more robust. Among the drugs studied for bipolar depression, acetylsalicylic acid, pioglitazone, memantine, and inositol were not superior to the placebo. The efficacy of the rest was superior to the placebo on at least one outcome measure, but the efficacy of modafinil/armodafinil was more robust than the others.

Clearly, drug repurposing in psychiatry has shown promise and challenges [40]. Compared to new drug development, drug repurposing will reduce the risk and cost of new treatments for mood disorders. However, it is very unlikely that all repurposed drugs will be effective in reducing depressive symptoms in MDD or bipolar depression. Some medications targeting mitochondrial function, oxidative stress balance, insulin resistance and/or anti-inflammatory pathways have demonstrated an antidepressant effect and/or cognitive benefits in patients with a mood disorder [41,42,43,44,45]. High rates of co-occurrent diabetes mellitus (DM) with bipolar or unipolar depression [46,47], and anhedonia [48,49] in patients with DM, suggested that insulin resistance or lack of insulin may play a role in the pathophysiology of depression [50,51,52,53,54]. That the risk of depression doubled in people with DM type 2 (DM2) compared to those without it further supports the role of insulin resistance in depression [55,56]. The risk of DM2 is also almost doubled (1.6 times greater) in people with bipolar disorder [57,58]. The weakened efficacy of lithium treatment in mood disorder due to the impairment of insulin signaling supports the importance of insulin in the treatment of depression [59]. Some recent studies indicated that depressive symptoms in bipolar disorder and MDD can be improved by some antihyperglycemic agents through insulin sensitization [43,60,61]. The effect of antidiabetics in both bipolar depression and MDD suggests that these two disorders may share some pathological commonalities when they are in depressive phase of their illness [44].

In addition to DM, there is also a bidirectional association between insulin resistance and depression [44,58,62,63]. Patients with a diagnosis of depression have an increased risk of insulin resistance [64,65,66]. Conversely, the presence of insulin resistance increases the likelihood of developing depression and the severity of depression [63,67]. The relationship between the treatment response and the level of insulin resistance supports the involvement of insulin in the pathophysiology of MDD [44].

The bidirectional association between insulin resistance and bipolar disorder was also found in recent clinical studies [68,69,70]. A higher relative risk of metabolic syndrome was also observed in people with bipolar disorders [71]. Bipolar patients with insulin resistance or DM2 showed severer clinical symptoms, greater cognition impairment and less improvement on mood stabilizer treatment compared to those without DM2 or impaired insulin resistance [59,69,72,73].

These data suggested that patients with a mood disorder, especially during the phase of depression, may have impaired insulin system and using antidiabetic agents to increase insulin activity may improve depressive symptoms [43,69,74,75,76,77]. However, some studies did not support this observation [78,79,80,81,82].

In previous reviews, the potential use of antihyperglycemic agents as antidepressants was noticed [74,83,84,85,86]. Since then, more double-blind RCTs and open-label studies were published [60,75,76,87,88,89,90,91]. A more current meta-analysis is necessary to provide guidance for future studies in this area. The aim of this analysis was to use double-blind RCTs to examine the efficacy and safety of antidiabetic agents in the treatment of MDD or bipolar depression.

## 2. Materials and Methods

### 2.1. Registration of Protocol

The protocol for this meta-analysis was registered at the International Platform of Registered Systematic Review and Meta-Analysis Protocols with the registration number INPLASY202090058. This meta-analysis was carried in accordance with the Preferred Reporting Items for Systematic Reviews and Meta-Analyses (PRISMA) guidelines.

### 2.2. Search Strategy

Three electronic databases, PubMed, Embase, and the Cochrane Library, and three clinical trial registry websites (clinicaltrials.gov, accessed on 9 December 2023, ISRCTN registry, and ICTRP registry) were searched with the language restricted to English and Chinese by two independent reviewer groups (RS and YC, a two-reviewer group, and JZ a one-person group). We also searched the gray literature for unpublished studies. The keywords “depression”, “bipolar disorder”, “hypoglycemic agents” and “randomized controlled trial” and relevant Mesh words and text words were connected using Boolean operators to identify eligible studies published up to October 2023. A manual search of the references in relevant articles was also conducted as a supplemental approach. The detailed search strategy and search results are presented in Appendix A (Table A1).

### 2.3. Inclusion/Exclusion Criteria

The inclusion criteria were defined as follows: (1) studies including subjects who had major depressive disorder or bipolar disorder diagnosed with the Diagnostic and Statistical Manual of Mental Disorders (DSM) or the International Classification of Diseases (ICD) or other standard diagnosis criteria; and (2) studies that were randomized, double-blind studies of an antidiabetic medication versus a placebo, either as monotherapy or adjunctive therapy, in the treatment for an acute depressive episode. A double-blind randomized crossover trial was also eligible, but only data in the first phase were included in the analysis. (3) Depressive severity was measured with at least one standardized depressive symptom rating scale.

The exclusion criteria were defined as follows: (1) studies that included patients who were not diagnosed with a mood disorder; (2) studies that did not have complete data to calculate the effect size of efficacy as a change in the depression rating score from baseline to endpoint, remission rate or response rate; (3) duplicated reports or secondary analyses of the same dataset of an eligible study; and (4) animal studies or case reports.

### 2.4. Study Selection and Data Extraction

The studies were screened and selected by two independent groups (RS and YC, and JZ), and the data were extracted by two independent researchers (BP, JZ) based on the pre-defined protocol following the PRISMA guidelines. Disagreements were resolved through discussion or by the third researcher (XY). The missing data that were essential for efficacy and safety, such as the mean and standard deviation of depression scales at baseline or endpoint, were obtained through re-calculation based on the reported data in the original publications, and only relevant formulas recommended in the Cochrane handbook were used [92]. In addition, contacting the corresponding author of a study was attempted to obtain missing data.

### 2.5. Assessment of Risk for Bias

The same two independent researchers (BP and JZ) independently assessed the risk for bias for each included study using the Cochrane tool (RoB 2.0). The bias of evidence was assessed relating to the primary outcomes, which compared the difference in the change in the depression rating score between the antidiabetics and placebo in each individual study. The biases being evaluated included selection bias, randomization and allocation concealment, performance bias, detection bias, attrition bias, reporting bias and other biases. Each bias domain included specific items, which were ranked as high, low or unclear. Disagreements were resolved through discussion or by the third researcher (XY).

### 2.6. Statistical Analysis

The efficacy outcomes included changes in the depression rating scale scores from baseline to the end of the study, response rate and remission rate. The changes in the depression scores from baseline to endpoint were used as the primary outcome. The response rate and remission rate were used as the secondary efficacy outcomes. In addition, the discontinuation rate was used as the tolerability outcome. Adverse events such as digestive symptoms, headaches, sexual disfunction, dry mouth, insomnia, and dizziness were used as safety outcomes.

The statistical analysis was performed using Review Manager software (version 5.3). The results were presented as the standardized mean difference (SMD) for continuous variables and the risk ratio (RR) for dichotomous data. Only a random effect model with 95% confidence intervals (CI) was used due to the expected heterogeneity from different interventions and diagnoses.

Heterogeneity was examined using the I^2^ values or Q statistics. I^2^ values of 0%, 25%, 50%, and 75% were indicative of no, low, moderate, and high heterogeneity, respectively [93]. Sensitivity analysis was conducted to examine the potential sources of heterogeneity when the I2 values were ≥50%. A fixed-effect model was used if the I^2^ statistic was less than 50%. Otherwise, a random-effect model was used.

Subgroup analyses were performed to address the heterogeneity based on region (North America vs. Middle East), medications (pioglitazone vs. metformin vs. insulin), and diagnosis (MDD vs. bipolar depression). Sensitivity analyses were performed by excluding studies with significantly different characteristics. We carried out sensibility testing for each pooled result with heterogeneity by performing step-by-step elimination analysis through removing one study at a time to address the possible resource of heterogeneity. A funnel plot was generated to assess the likelihood of publication bias in the primary results, and Egger’s tests were performed as an aid when more than ten studies were available in a pool [94].

## 3. Results

### 3.1. Search Results

**Figure 1:**
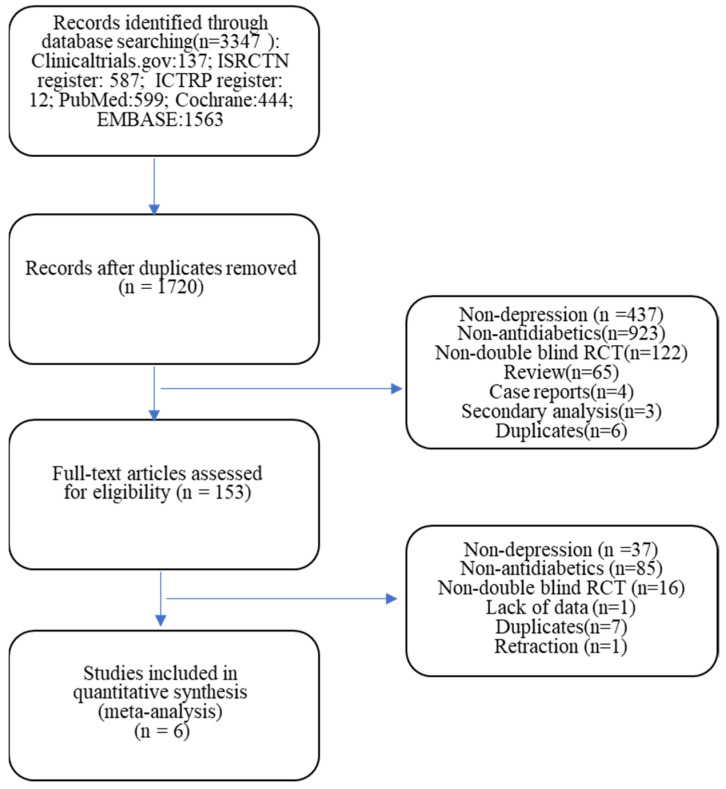
Flowchart.

The electronic search uncovered 3347 records. After removing duplicates, 1720 remained. After reading the title and abstract, 153 were eligible for full text examination. After reading the full text of 153 publications, 7 studies [75,76,80,81,82,87,88] met the criteria for the meta-analysis. However, one of the seven studies [75] was excluded from the final analysis because the publication associated with the study had been retracted by the journal due to some concerns about the data presented. One of the six studies had a crossover design [81], but only the data from the first phase of the study were included in the analysis. The study selection process is outlined in Figure 1.

### 3.2. Characteristics of the Eligible Studies

Six studies with 399 patients (201 in active treatment, 198 in placebo), a mean sample size of 67 for each study, were eligible for the meta-analysis. All the included studies were double-blind, randomized, and placebo-controlled trials. Five of the six trials were two-armed, with an adjunctive antidiabetic versus an adjunctive placebo to ongoing treatment. One trial [80] had a four-armed design comparing adjunctive pioglitazone versus adjunctive placebo in the insulin resistance or insulin sensitive group.

In terms of diagnoses, three studies only included MDD participants [76,81,87], two studies only included patients with bipolar depression [82,88], and one study included both MDD and bipolar depression patients [80]. As for the medications used as an antidiabetic, four studies used pioglitazone tablets [80,82,87,88], one studies used metformin tablets [76], and one study used intranasal insulin [81].

In terms of using depression rating scales, three studies used the Hamilton Depression Rating Scale (HDRS) [80,87,88], two studies used the Montgomery–Asberg Depression Rating Scale (MADRS) [81,82] and one study used the Inventory of Depressive Symptomatology (IDS). The detailed characteristics of each study are outlined in Table 1. 

### 3.3. Risk of Bias in the Included Studies

**Figure 2:**
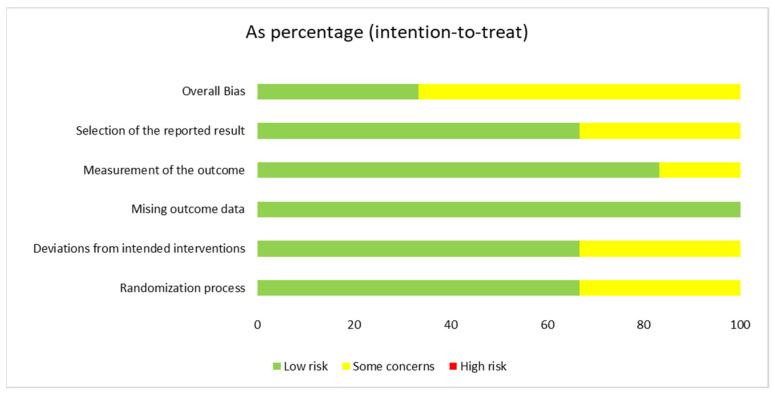
Risk of bias assessed with Version 2 of the Cochrane risk-of-bias tool for randomized trials (RoB 2.0).

Most studies were at a low risk for all the domains of potential biases. However, two studies only reported limited information on the generation of the randomization sequence and allocation concealment [76,82]. The assessments of bias are summarized in Figure 2.

### 3.4. Primary Outcomes

**Figure 3:**
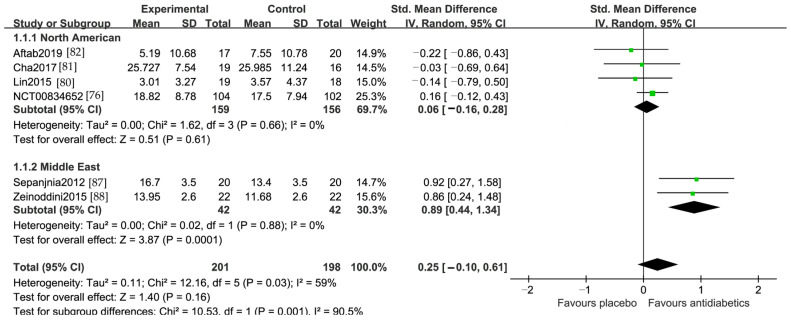
Forest plot of changes of the depressive rating score from baseline to endpoint comparing antidiabetics vs. placebo: Subgroup analysis by region [76,80,81,82,87,88].

Of the 399 patients included in this meta-analysis, the SMD was 0.25 (95% CI −0.1, 0.61). The studies were heterogeneous, with an I^2^ value of 59% (Figure 3). Neither a funnel plot nor Egger’s test was performed because less than 10 studies were meta-analyzed in each pool.

**Figure A1:**
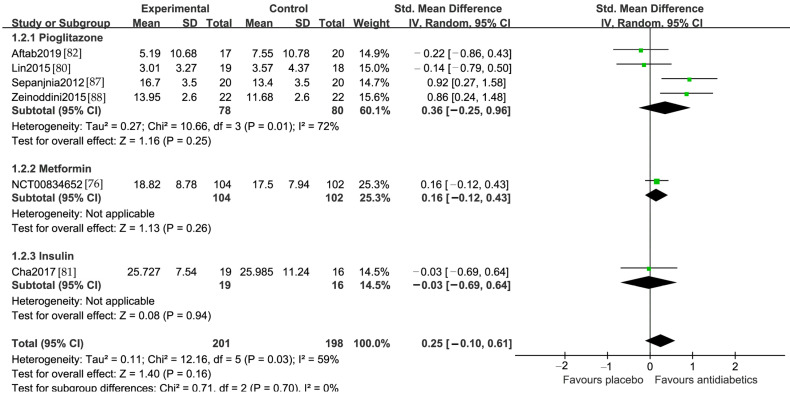
Forest plot of changes in the depressive rating score from baseline to endpoint comparing antidiabetics vs. placebo: Subgroup analysis by medication [76,80,81,82,87,88].

The subgroup analyses according to the region indicated that the regional difference was a source of heterogeneity. Analysis of two studies from the Middle East showed a significant reduction in depressive symptom severity in the antidiabetic adjunctive arm compared to that in the placebo adjunctive arm. The SMD was 0.89 (95% CI 0.44, 1.34) and I^2^ = 0%. However, there was no significant difference in changes in the depression scores in the four studies from North America, with an SMD of 0.06 (95% CI −0.16, 0.28) and I^2^ = 0% (Figure 3). The subgroup analyses on the medications (pioglitazone, metformin and insulin) and diagnosis (MDD and bipolar depression) did not detect any clinical relevance of heterogeneity (Appendix A, Figure A1). Sensitive analysis performed by individually removing each study indicated that no single study notably affected the overall *p* value for heterogeneity. Neither a funnel plot nor Egger’s test was performed because less than 10 studies were meta-analyzed in each pool.

### 3.5. Response Rate

**Figure 4:**
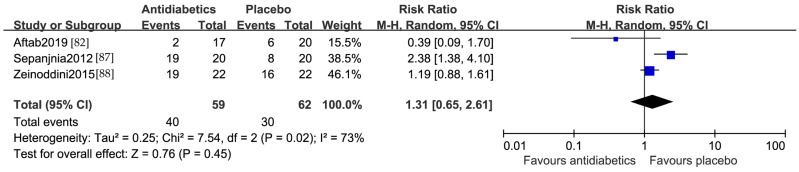
Forest plot of the response rate comparing antidiabetics vs. placebo [82,87,88].

A total of 121 patients from two studies from the Middle East [87,88] and one from North America [82] were included in the responder analysis. The pooled effects size of the response rates was not significantly different between the active treatment and placebo, with an RR of 1.31 (95% CI 0.65, 2.61). Heterogeneity among the included studies was detected (I^2^ = 73%) (Figure 4). Sensitive analysis performed by removing one study at a time indicated all the results remained consistent. Since only three studies were included, an examination for publication bias was not performed.

### 3.6. Remission Rate

**Figure 5:**
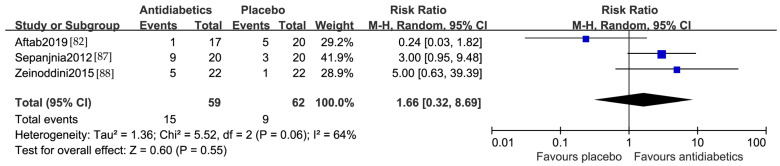
Forest plot of the remission rate comparing antidiabetics vs. placebo [82,87,88].

The same three studies that reported data on the remission rate were pooled into a meta-analysis. The effects size was nonsignificant, with an RR of 1.66 (95% CI 0.32, 8.69) and I^2^ = 64% (Figure 5). Since only three studies were included, an examination for publication bias was not performed.

Sensitive analysis performed by individually removing each study indicated all the results remained consistent, with one exception [82]. The Aftab 2019 study was the only study from North America that was included in the meta-analysis, and the difference between the treatment and placebo arms became statistically significant after removal of this study, with an RR of 3.39 (95% CI 1.24, 9.25) and I^2^ = 0%.

### 3.7. Tolerability and Safety Outcomes

**Figure 6:**
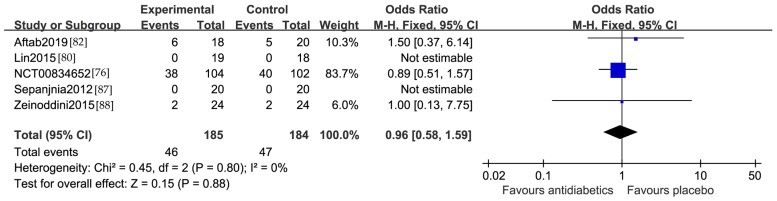
Dropout rate comparing antidiabetics vs. placebo [76,80,82,87,88].

Five studies reported data on the dropout rate [76,80,82,87]. The results indicated there was no significant difference in the discontinuation rate between the treatment arm (n = 185) and the placebo arm (n = 184), with an RR of 0.96 (95% CI 0.58, 1.59) and I^2^ = 0% (Figure 6). A sensitivity analysis performed by removing one study at a time indicated that no single study had a significant effect on the heterogeneity and the overall results.

**Figure 7:**
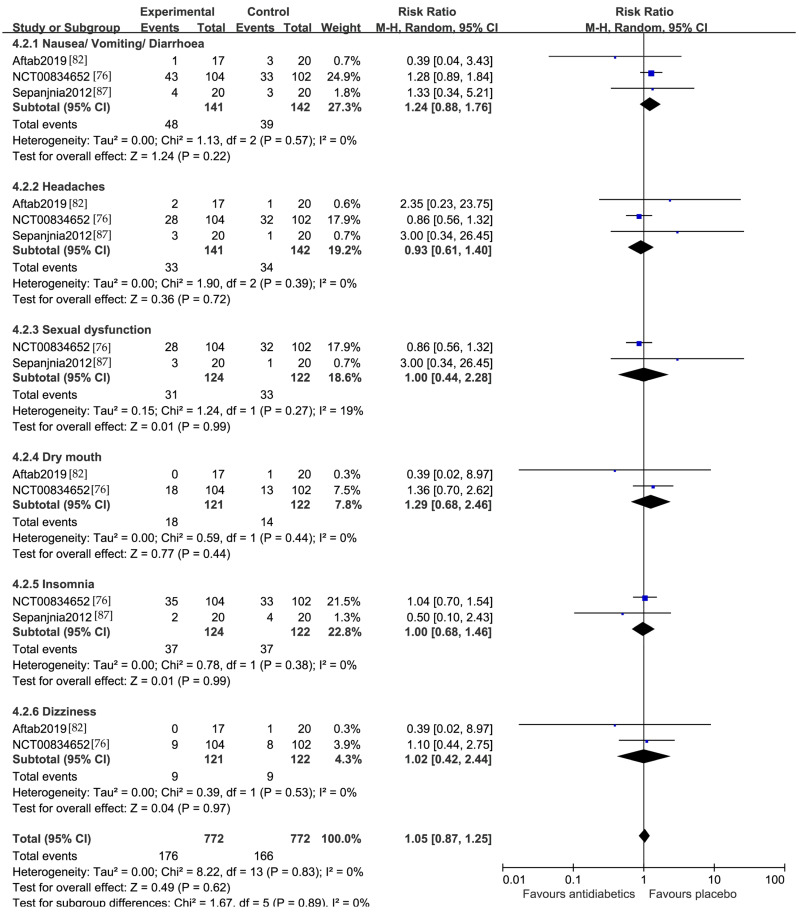
Side effects comparing antidiabetics vs. placebo [76,82,87].

Three studies reported adverse events in the gastrointestinal tract (nausea/vomiting/diarrhea) and headaches [76,82,87]. There was no significant difference between the active arm (n = 141) and the placebo (n = 142) in terms of the gastrointestinal side effects, with an RR of 1.24 (95% CI 0.88, 1.76), and headaches, with an RR of 0.93 (95% CI 0.61, 1.40), respectively. Two studies reported sexual dysfunction and insomnia [76,87]. There was no significant difference between the active arm (n = 124) and the placebo (n = 122) in terms of sexual dysfunction, with an RR of 1.00 (95% CI 0.44, 2.28), and insomnia, with an RR of 1.00 (95% CI 0.68, 1.46), respectively. Two studies reported dry mouth and dizziness [76,82]. There was also no significant difference between the active arm (n = 121) and the placebo (n = 122) in terms of dry mouth, with an RR = 1.29 (95% CI 0.68 to 2.46), and dizziness, with an RR of 1.02 (95% CI 0.42, 2.44), respectively (Figure 7). The heterogeneity for all these side effects was 0%.

## 4. Discussion

In this meta-analysis, we did not find any significant difference of active antidiabetic medication over placebo in the acute treatment of a major depressive episode of MDD or bipolar depression. However, subgroup and sensitivity analyses found that antidiabetics were significantly superior to placebo in reducing depressive symptoms in studies from the Middle East, but not in studies from North America. Overall, antidiabetics were as safe and well tolerated as placebo.

The insignificant difference of active antidiabetic medication over placebo in the current meta-analysis is inconsistent with a previous meta-analysis on the efficacy of pioglitazone versus placebo or metformin in the acute treatment of a major depressive episode [95]. In this earlier meta-analysis, four RCTs [79,80,87,88] were included. Three of the four studies [80,87,88] were also included in our analysis. The one not included in our analysis [79] was a study comparing pioglitazone versus metformin in a major depressive episode of a mood disorder. This earlier meta-analysis found that pioglitazone was superior to placebo in improving the remission rate and depressive symptoms. The discrepancy between our analysis and the earlier analysis might be due to the difference in the number of studies and what studies were included, and/or regional differences as we found in our analysis. The earlier analysis included three studies from the Middle East [79,87,88] that were similar to our current analysis, and all three studies showed a significant improvement in depression with pioglitazone compared to placebo. The one study that did not find a significant improvement in depression with pioglitazone over placebo was from the United States [80]. In our current analysis, two studies were from the Middle East and three were from North America (Table 1). All two studies from the Middle East showed that antidiabetics were significantly superior to placebo in reducing depression symptoms, but none of all four studies from North America found significant differences between antidiabetics and placebo (Figure 3). Sensitive analysis of the remission rates with pioglitazone versus placebo, including two studies from the Middle East [87,88] and one study from North America [82], also found regional differences (Figure 5).

It remains unclear if the efficacy difference of antidiabetics in reducing depressive symptoms from these two regions was due to the difference in biology, culture, lifestyles, diets, behaviors or the inclusion criteria of the study subjects. In a systematic review of the efficacy, tolerability and safety of atypical antipsychotics in the treatment of bipolar patients from Asia and North America [96], we found significant differences between the two groups of patients in terms of discontinuation due to adverse events and some common side effects. However, the efficacy of antipsychotics in acute mania or bipolar depression was similar between the two groups. Since those studies were sponsored by pharmaceutical companies and used the same study designs in both regions, the difference between the two groups of patients were likely because of the difference in biology, cultures and behaviors. Although, in our current analysis, each study was designed and conducted by each group of researchers, the difference in biology, cultures and behaviors could still play a role in the efficacy difference of the same antidiabetic in different studies for the treatment of depression. A racial disparity in the efficacy of antidepressant in major depression [97,98] and differential associations between depressive symptoms and obesity among African Americans and Caucasian Americans [99] might also help to explain the regional differences in our analysis. In addition, the two studies from the Middle East excluded patients with metabolic syndrome, which might also affect the antidepressant effect from antidiabetics.

It is well known that diets play an important role in the treatment of both diabetic mellitus and depression [100,101,102,103,104,105]. A healthy dietary pattern was associated with a reduction of depression in patients with diabetes [106]. A case control study found that a low protein diet regimen 6 days a week could significantly decrease depressive symptoms in type 2 diabetic patients [107].

The associations between diets and systemic inflammation, and between diets and behaviors, have been studied. A United Kingdom biobank study found that low-grade inflammation was a mediator between diet and behavioral disinhibition [108]. It is reported that the prudent diet was negatively, and the meat-based diet, such as the Western style food, was positively associated with several pro-inflammatory biomarkers [109]. It is also reported that the insulin insensitivity caused by pro-inflammatory cytokines may result in metabolic abnormalities that are associated with depression [61]. Moreover, pro-inflammatory cytokines may directly cause depressive symptoms [47]. A bidirectional relationship between depression and inflammation has been supported by a large number of studies [110,111]. Importantly, a number of studies support the anti-inflammatory effect of a Mediterranean diet [112]. Antidiabetics also have anti-inflammatory effect [113]. However, the results of our meta-analysis suggest that the anti-inflammatory effect of antidiabetics alone may not be enough to cause significant differences between an antidiabetic and placebo in reducing depressive symptoms. The positive finding from the Middle East studies and negative finding from North American studies suggest that different diets may create different conditions for antidiabetics to produce an antidepressant effect in MDD or bipolar depression. The condition for the antidepressant effect of antidiabetics might be the level inflammation, i.e., no inflammation or low-level of inflammation.

Different diet patterns and food structures between countries in the North America and the Middle East areas were reported [114,115]. Therefore, it is reasonable to speculate that the differences in diets between the two geographic areas might contribute to the regional difference in the results in the current analysis. The positive studies from the Middle East might be due to the diets that the patients consumed because they are geographically close to The Mediterranean Sea and subsequently had no inflammation or low-level inflammation. This speculation can be studied by measuring the level of inflammation in patients from this region in future studies. Clearly, the role of diets in the efficacy of antidiabetic agents in the acute treatment of depression is worthy of further exploration.

The metabolic status could also potentially affect the efficacy of antidiabetics in reducing depressive symptoms. Open-label studies found that rosiglitazone and pioglitazone were able to reduce depressive symptoms in patients with major depression comorbid insulin resistance, metabolic syndrome, or DM2 [60,116]. The antidepressant effect of antidiabetics might occur through their insulin-sensitizing effect, especially in patients with metabolic disturbances [61,117,118]. Of the six studies included in the current meta-analysis, two studies required depressive patients with a BMI of 18.5–40 kg/m^2^ [80] or greater than 28 [76], and two studies excluded patients with metabolic syndrome [87,88]. All the two studies that excluded patients with metabolic syndrome showed that an antidiabetic was significantly superior to the placebo in reducing depressive symptoms (Figure 3). This suggests that patients from the Middle East and those who do not have metabolic syndrome may benefit from adding an antidiabetic medication to their ongoing medications. Future studies with uniform inclusion criteria may help us to determine the efficacy of antidiabetics in the treatment of MDD or bipolar depression with different metabolic burdens.

Importantly, all the studied antidiabetic agents in the current analysis were safe and tolerated as well as the placebo. The side effects, including headaches, sexual dysfunction, dry mouth, insomnia, and dizziness, were not significantly different from the placebo in the current meta-analysis, which suggests that an antidiabetic adjunctive therapy to an ongoing treatment of a major depressive episode is very unlikely to cause an additional severe side effect burden to the ongoing treatment(s). However, it should be kept in mind that the relatively benign side effects of the antidiabetics in the currently reviewed studies could be due to a relatively short duration of the studies and/or relatively lower dosages of antidiabetic agents compared to doses in the treatment of DM (Table 1).

Although the overall result of this meta-analysis did not find a significant difference between antidiabetics and placebo in reducing depressive symptoms, the results from the subgroup analysis suggested that depressed patients from the Middle East or those without metabolic syndrome may benefit from an antidiabetic for their depressive symptoms. More studies are needed to verify the efficacy of antidiabetics in the treatment of depression. Meanwhile, antidiabetics can be considered for patients with a major depressive episode from the Middle East who are not fully responsive to traditional antidepressants and/or mood stabilizers and who do not have metabolic syndrome. The safety profiles and antidepressant effect in depressed patients with metabolic abnormality of antidiabetics suggest that the potential benefit of adding an antidiabetic to ongoing treatment(s) for patients who have depression and metabolic disturbance is an over potential risk. Therefore, antidiabetics can also be considered for patients with a major depressive episode and metabolic abnormalities.

As mentioned in the Introduction section, antidiabetic agents were among the drugs studied for the treatment of MDD and bipolar depression [38,39]. The mechanism of the antidepressant effect of antidiabetics is believed to act through improving insulin resistance and reducing inflammation, mitochondria abnormalities, oxidative stress [38]. The overall impression was that pioglitazone was superior to placebo in MDD [38], but not in bipolar depression [39]. The result of pioglitazone in bipolar depression was based on a meta-analysis of pioglitazone [119], which included only two RCTs of pioglitazone in bipolar depression [82,88]. The two RCTs were also included in the current meta-analysis, one from the Middle East with positive results and another from North America with negative results. The results of this previous meta-analysis with two RCTs were consistent with the results of our current meta-analysis. Overall, we still cannot draw a firm conclusion on the clinical utility of repurposed pioglitazone or other antidiabetics as an adjunctive treatment for depression due to the inconsistent results and limited evidence. In contrast, repurposing modafinil/armodafinil, pramipexole, celecoxib and N-acetylcysteine for depression is seemingly more successful than the use of antidiabetics and other medications [38,39].

This analysis had several limitations. First, the sample size was small, and only 6 studies with a total of 399 patients met our criteria. With the exception of 1 study with 206 participants [76], most of other studies had a number of less than 44 patients. However, all six included studies were randomized, double-blind, placebo-control trials with a methodologically high quality. Second, although the high heterogeneity was addressed by the subgroup analysis and the study origins were identified as the source of heterogeneity, the diagnostic criteria for a depressive episode in mood disorder (MDD or bipolar depression), comorbidities, concomitant psychotropic treatments, and study duration were also heterogeneous. We were unable to address those heterogeneities in the current analysis. Third, we only explored the efficacy and safety of antidiabetic agents in an acute depressive episode with durations of 4–16 weeks. The long-term efficacy and safety of antidiabetics in depression remain unclear. Fourth, all the studies were either from North America or the Middle East. The results may be not applied to other regions and populations. However, despite these considerable limitations, this meta-analysis has provided updated information on the efficacy and safety of antidiabetic agents in depression based on studies with high-quality study designs and low publication bias.

## 5. Conclusions

The current meta-analysis does not support the efficacy of antidiabetics in the treatment of unipolar and bipolar depression. However, the subgroup analysis indicates that patients from the Middle East who do not have metabolic syndrome may benefit from adding an antidiabetic medication to their ongoing medication(s). Antidiabetic agents appear safe and well tolerated in the treatment of an acute depressive episode. Considering the high prevalence of metabolic syndrome and insulin resistance in patients with a mood disorder, the potential benefit of adding an antidiabetic is likely over the risk. Future large studies with uniform study designs are essential to determine the efficacy of antidiabetics in the acute treatment of MDD or bipolar depression.

## Figures and Tables

**Table 1:** Summary of the included placebo-controlled, double-blind, randomized studies on antidiabetics in depression treatment.

Study, Year	Region	Female N (%)	Diagnosis Instrument	Diagnosis	TR Status	Duration (Weeks)	Efficacy Measure	Active Medication/ Max Dosage	Concomitant	Outcomes	Rank of ROB
Aftab, 2019 [82]	USA	24 37 (64.9%)	DSM-IV-TR	BP-I, II, NOS	No	8	MADRS	Pioglitazone 45 mg	MS/APS/ADS/BZD	No statistically significant difference was found in the response rates, remission rates and score change from baseline between the activator and placebo	Low
Cha, 2017 [81]	Canada	22 35 (62.9%)	DSM-IV-TR	MDD	Yes	4	MADRS	Intranasal Insulin1.6 mL	Not specified	No statistically significant difference was found in the improvements on the MADRS between the activator and placebo	Low
Lin, 2015 [80]	USA	29 37 (78.4%)	DSM-IV-TR	MDD or BP I, II or NOS	No	12	HDRS	Pioglitazone 30 mg	Not specified	No statistically significant difference was found in the decline of HDRS-21 between the activator and placebo	Low
NCT00834652 [76]	USA	171 206 (83.0%)	DSM-IV-TR	MDD, BMI > 28.7	No	16	BDI	Metformin 1000 mg	Sertraline	Sertraline plus metformin had a significant difference in the score change from baseline compared to the placebo	Low
Sepanjnia, 2012 [87]	Iran	29 40 (72.5%)	DSM-IV-TR	MDD	No	6	HDRS	Pioglitazone 30 mg	Citalopram	The activator had a significant difference in the response rates, remission rates and score change from baseline compared to the placebo	Low
Zeinoddini, 2015 [88]	Iran	15 44 (34.1)	DSM-IV-TR	BP-I	No	6	HDRS	Pioglitazone 30 mg	Lithium	The activator had a significant difference in the score change from baseline compared to the placebo, but not in the response rates or remission rates.	Low

Abbreviation: BP = bipolar disorder; MDD = major depression; N = number.

## Data Availability

Data are contained within the article.

## Appendix A

**Table A1:** The detailed search strategy and results of the first search of PubMed.

Search	#3 AND #6 AND #9	496
	Search: (((Hypoglycemic*[Title/Abstract] OR Antidiabetic*[Title/Abstract] OR Acarbose[Title/Abstract] OR acarviosine-glucose[Title/Abstract] OR Acetohexamide[Title/Abstract] OR Allicin[Title/Abstract] OR alliin[Title/Abstract] OR alogliptin[Title/Abstract] OR anagliptin[Title/Abstract] OR bexagliflozin[Title/Abstract] OR Biguanides[Title/Abstract] OR Biphasic Insulins[Title/Abstract] OR BRL 26830A[Title/Abstract] OR Buformin[Title/Abstract] OR Butoxamine[Title/Abstract] OR butyl isobutyl phthalate[Title/Abstract] OR Canagliflozin[Title/Abstract] OR Carbutamide[Title/Abstract] OR castanospermine[Title/Abstract] OR Chlorpropamide[Title/Abstract] OR ciglitazone[Title/Abstract] OR CM-10-18 compound[Title/Abstract] OR conduritol B[Title/Abstract] OR deoxyvalidamine[Title/Abstract] OR dihydroacarbose[Title/Abstract] OR diphenyleneiodonium[Title/Abstract] OR diprotin A[Title/Abstract] OR dorsilurin F[Title/Abstract] OR dulaglutide[Title/Abstract] OR emeriamine[Title/Abstract] OR empagliflozin[Title/Abstract] OR englitazone[Title/Abstract] OR etomoxir[Title/Abstract] OR Exenatide[Title/Abstract] OR fenugreek seed meal[Title/Abstract] OR firuglipel[Title/Abstract] OR ginnalin A[Title/Abstract] OR glibornuride[Title/Abstract] OR Gliclazide[Title/Abstract] OR glimepiride[Title/Abstract] OR Glipizide[Title/Abstract] OR gliquidone[Title/Abstract] OR Glyburide[Title/Abstract] OR Gusperimus[Title/Abstract] OR inositol phosphate glycan[Title/Abstract] OR Insulin[Title/Abstract] OR Ipragliflozin[Title/Abstract] OR Islet Amyloid Polypeptide[Title/Abstract] OR iso-oenothein C[Title/Abstract] OR isoacarbose[Title/Abstract] OR jamutannin A[Title/Abstract] OR jamutannin B[Title/Abstract] OR lamesticumin G[Title/Abstract] OR Linagliptin[Title/Abstract] OR lipoyl vildagliptin[Title/Abstract] OR Liraglutide[Title/Abstract] OR Lixisenatide[Title/Abstract] OR Lonchocarpene[Title/Abstract] OR meglitinide[Title/Abstract] OR Metformin[Title/Abstract] OR midaglizole[Title/Abstract] OR miglitol[Title/Abstract] OR miglustat[Title/Abstract] OR mitiglinide[Title/Abstract] OR MK-2640[Title/Abstract] OR MR1704[Title/Abstract] OR Nateglinide[Title/Abstract] OR nigrosporamide A[Title/Abstract] OR NN 7201[Title/Abstract] OR Peroxovanadate[Title/Abstract] OR Phenformin[Title/Abstract] OR phenyl biguanide[Title/Abstract] OR Pioglitazone[Title/Abstract] OR pistagremic acid[Title/Abstract] OR polyflavanostilbene A[Title/Abstract] OR ponalrestat[Title/Abstract] OR pradimicin Q[Title/Abstract] OR pramlintide[Title/Abstract] OR purunuside A[Title/Abstract] OR purunuside B[Title/Abstract] OR purunuside C[Title/Abstract] OR pycnalin[Title/Abstract] OR remogliflozin etabonate[Title/Abstract] OR repaglinide[Title/Abstract] OR Rosiglitazone[Title/Abstract] OR rubasperone A[Title/Abstract] OR rubasperone B[Title/Abstract] OR S009-0629[Title/Abstract] OR salicortin[Title/Abstract] OR saxagliptin[Title/Abstract] OR schulzeine A[Title/Abstract] OR scirpusin C[Title/Abstract] OR sergliflozin etabonate[Title/Abstract] OR Sitagliptin Phosphate[Title/Abstract] OR Sulphostin[Title/Abstract] OR Syrrx 106124[Title/Abstract] OR tirzepatide[Title/Abstract] OR Tolazamide[Title/Abstract] OR Tolbutamide[Title/Abstract] OR Topiramate[Title/Abstract] OR Trestatin[Title/Abstract] OR Troglitazone[Title/Abstract] OR valienamine[Title/Abstract] OR vanadyl sulfate[Title/Abstract] OR velagliflozin[Title/Abstract] OR Vildagliptin[Title/Abstract] OR Voglibose[Title/Abstract] OR Volagidemab[Title/Abstract] OR Xultophy[Title/Abstract] OR zopolrestat[Title/Abstract]) OR ((((((((((((((((((((((((((((((((((((((((((((((((((((((((((((((((((((((“zopolrestat” [Supplementary Concept]) OR (“Xultophy” [Supplementary Concept])) OR (“volagidemab” [Supplementary Concept])) OR (“voglibose” [Supplementary Concept])) OR (“Vildagliptin”[Mesh])) OR (“velagliflozin” [Supplementary Concept])) OR (“vanadyl sulfate” [Supplementary Concept])) OR (“valienamine” [Supplementary Concept])) OR (“Troglitazone”[Mesh])) OR (“trestatin” [Supplementary Concept])) OR (“Topiramate”[Mesh])) OR (“Tolbutamide”[Mesh])) OR (“Tolazamide”[Mesh])) OR (“TMC 2C” [Supplementary Concept])) OR (“TMC 2B” [Supplementary Concept])) OR (“tirzepatide” [Supplementary Concept])) OR (“Syrrx 106124” [Supplementary Concept])) OR (“sulphostin” [Supplementary Concept])) OR (“Sitagliptin Phosphate”[Mesh])) OR (“sergliflozin etabonate” [Supplementary Concept])) OR (“schulzeine A” [Supplementary Concept])) OR (“saxagliptin” [Supplementary Concept])) OR (“salicortin” [Supplementary Concept])) OR (“S009-0629” [Supplementary Concept])) OR (“rubasperone B” [Supplementary Concept])) OR (“rubasperone A” [Supplementary Concept])) OR (“Rosiglitazone”[Mesh])) OR (“repaglinide” [Supplementary Concept])) OR (“remogliflozin etabonate” [Supplementary Concept])) OR (“pycnalin” [Supplementary Concept])) OR (“purunuside C” [Supplementary Concept])) OR (“purunuside B” [Supplementary Concept])) OR (“purunuside A” [Supplementary Concept])) OR (“pramlintide” [Supplementary Concept])) OR (“pradimicin Q” [Supplementary Concept])) OR (“ponalrestat” [Supplementary Concept])) OR (“polyflavanostilbene A” [Supplementary Concept])) OR (“pistagremic acid” [Supplementary Concept])) OR (“Pioglitazone”[Mesh])) OR (“phenyl-6-deoxy-6-(morpholin-4-yl)glucopyranoside” [Supplementary Concept])) OR (“phenyl biguanide” [Supplementary Concept])) OR (“Phenformin”[Mesh])) OR (“peroxovanadate” [Supplementary Concept])) OR (“NN 7201” [Supplementary Concept])) OR (“nigrosporamide A” [Supplementary Concept])) OR (“Nateglinide”[Mesh])) OR (“N-phenyl-4,5,6,7-tetrachlorophthalimide” [Supplementary Concept])) OR (“N-nonyl-1-deoxynojirimycin” [Supplementary Concept])) OR (“N-alanylproline-O-(4-nitrobenzoyl)hydroxylamine” [Supplementary Concept])) OR (“MYL-1501D” [Supplementary Concept])) OR (“MR1704” [Supplementary Concept])) OR (“MK-2640” [Supplementary Concept])) OR (“mitiglinide” [Supplementary Concept])) OR (“miglustat” [Supplementary Concept])) OR (“miglitol” [Supplementary Concept])) OR (“midaglizole” [Supplementary Concept])) OR (“methyl 2-tetradecylglycidate” [Supplementary Concept])) OR (“methionyl-2-cyanopyrrolidine” [Supplementary Concept])) OR (“meglitinide” [Supplementary Concept])) OR (“lonchocarpene” [Supplementary Concept])) OR (“lixisenatide” [Supplementary Concept])) OR (“Liraglutide”[Mesh])) OR (“lipoyl vildagliptin” [Supplementary Concept])) OR (“Linagliptin”[Mesh])) OR (“lamesticumin G” [Supplementary Concept])) OR (“jamutannin B” [Supplementary Concept])) OR (“jamutannin A” [Supplementary Concept])) OR (“isoacarbose” [Supplementary Concept])) OR ((((((((((((((((((((((((((((((((((((((((((((((((((((((((((((((((((((((((((((((((((((((((“(2S,3R,4R,5S,6R)-2-(4-chloro-3-(4-ethoxybenzyl)phenyl)-6-(methylthio)tetrahydro-2H-pyran-3,4,5-triol” [Supplementary Concept]) OR (“(3,3-difluoropyrrolidin-1-yl)-(4-(4-pyrimidin-2-yl-piperazin-1-yl)-pyrrolidin-2-yl)methanone” [Supplementary Concept])) OR (“1,4-dideoxy-1,4-((2,3,4,5,6-pentahydroxyhexyl)episulfoniumylidene)arabinitol” [Supplementary Concept])) OR (“1-(((1-(hydroxymethyl)cyclopentyl)amino)acetyl)pyrrolidine-2,5-dicarbonitrile” [Supplementary Concept])) OR (“1-(((2-((5-cyanopyridin-2-yl)amino)ethyl)amino)acetyl)-2-cyano-(S)-pyrrolidine” [Supplementary Concept])) OR (“1-((6-hydroxy-2-azabicyclo(2.2.1)hept-3-yl)carbonyl)-2-pyrrolidinecarbonitrile” [Supplementary Concept])) OR (“1-(2’-amino-3’,3’-dimethylbutanoyl)pyrrolidine-2-carbonitrile” [Supplementary Concept])) OR (“1-(2-(5-cyano-2-pyridinylamino)ethylamino)acetyl-2-pyrrolidinecarbonitrile” [Supplementary Concept])) OR (“1-(4-aminomethyl-4-(3-chlorophenyl)cyclohexyl)-tetrahydropyrimidin-2-one” [Supplementary Concept])) OR (“13-hydroxykompasinol A” [Supplementary Concept])) OR (“2,3,4,5,6-pentagalloylglucose” [Supplementary Concept])) OR (“2,4-thiazolidinedione” [Supplementary Concept])) OR (“2-(3-(4-ethoxybenzyl)-4-chlorophenyl)-6-hydroxymethyltetrahydro-2H-pyran-3,4,5-triol” [Supplementary Concept])) OR (“2-bromopalmitate” [Supplementary Concept])) OR (“2-tetradecylglycidic acid” [Supplementary Concept])) OR (“24,25,26,27-tetranor-apotirucalla-(apoeupha)-1-senecioyloxy-3,7-dihydroxy-14,20,22-trien-21,23-epoxy” [Supplementary Concept])) OR (“3,5-dimethoxy-4’-O-prenyl-trans-stilbene” [Supplementary Concept])) OR (“3-amino-1-(1-boronic-ethyl)pyrrolidine-2-one” [Supplementary Concept])) OR (“3-amino-4-(2,4,5-trifluorophenyl)-*N*-(4-(6-(2-methoxyethoxy)benzothiazol-2-yl)tetrahydropyran-4-yl)butanamide” [Supplementary Concept])) OR (“3-amino-4-(3,3-difluoropyrrolidin-1-yl)-*N*,*N*-dimethyl-4-oxo-2-(4-(1,2,4)triazolo(1,5-a)pyridin-6-ylphenyl)butanamide” [Supplementary Concept])) OR (“3-O-digalloyl-1,2,4,6-tetra-O-galloylglucose” [Supplementary Concept])) OR (“4-(3-amino-4-(2,4,5-trifluorophenyl)butanoyl)-3-(2,2,2-trifluoroethyl)-1,4-diazepan-2-one” [Supplementary Concept])) OR (“4-(8-(4-fluorobenzyl)-3-(trifluoromethyl)-5,6-dihydro(1,2,4)triazolo(4,3-a)pyrazin-7(8H)-yl)-4-oxo-1-(2,4,5-trifluorophenyl)butan-2-amine” [Supplementary Concept])) OR (“4-(8-(4-fluorobenzyl)-3-(trifluoromethyl)-5,6-dihydro(1,2,4)triazolo(4,3-a)pyrazin-7(8H)-yl)-4-oxo-1-(2,4,5-trifluorophenyl)butan-2-amine” [Supplementary Concept] AND “4-(8-methyl-3-(trifluoromethyl)-5,6-dihydro(1,2,4)triazolo(4,3-a)pyrazin-7(8H)-yl)-4-oxo-1-(2,4,5-trifluorophenyl)butan-2-amine” [Supplementary Concept])) OR (“4-O-glucopyranosylmoranoline” [Supplementary Concept])) OR (“5,7-dihydroxy-6,8-dimethyl-4’-methoxyflavone” [Supplementary Concept])) OR (“5-amino-5-deoxygluconic acid delta-lactam” [Supplementary Concept])) OR (“6-((4-ethylphenyl)methyl)-3’,4’,5’,6’-tetrahydro-6’-(hydroxymethyl)spiro(isobenzofuran-1(3H),2’-(2H)pyran)-3’,4’,5’-triol” [Supplementary Concept])) OR (“6-amino-pyrido(2,3-d)pyrimidine-2,4-dione” [Supplementary Concept])) OR (“6-aminocyclohex-4-ene-1,2,3-triol” [Supplementary Concept])) OR (“6-methyl-3-hydroxypiperidine-2-carboxylic acid” [Supplementary Concept])) OR (“7-deoxycastanospermine” [Supplementary Concept])) OR (“8-(2-hydroxypropan-2-yl)-5-hydroxy-7-methoxy-6-methyl-4’-methoxyflavone” [Supplementary Concept])) OR (“8-hydroxy-2,2,14,14-tetramethylpentadecanedioic acid” [Supplementary Concept])) OR (“ABT 341” [Supplementary Concept])) OR (“Acarbose”[Mesh])) OR (“acarviosinyl-(1-9)-3-glucopyranosylpropene” [Supplementary Concept])) OR (“Acetohexamide”[Mesh])) OR (“AICA ribonucleotide” [Supplementary Concept])) OR (“allicin” [Supplementary Concept])) OR (“alliin” [Supplementary Concept])) OR (“alogliptin” [Supplementary Concept])) OR (“anagliptin” [Supplementary Concept])) OR (“bexagliflozin” [Supplementary Concept])) OR (“Biguanides”[Mesh])) OR (“Biphasic Insulins”[Mesh])) OR (“BRL 26830A” [Supplementary Concept])) OR (“Buformin”[Mesh])) OR (“Butoxamine”[Mesh])) OR (“butyl isobutyl phthalate” [Supplementary Concept])) OR (“Canagliflozin”[Mesh])) OR (“Carbutamide”[Mesh])) OR (“castanospermine” [Supplementary Concept])) OR (“Chlorpropamide”[Mesh])) OR (“ciglitazone” [Supplementary Concept])) OR (“CM-10-18 compound” [Supplementary Concept])) OR (“conduritol B” [Supplementary Concept])) OR (“copper bis(3,5-diisopropylsalicylate)” [Supplementary Concept])) OR (“deoxyvalidamine” [Supplementary Concept])) OR (“dihydroacarbose” [Supplementary Concept])) OR (“dihydropyrano(3,2-c)quinoline” [Supplementary Concept])) OR (“diphenyleneiodonium” [Supplementary Concept])) OR (“diprotin A” [Supplementary Concept])) OR (“disodium (R,R)-5-(2-((2-(3-chlorophenyl)-2-hydroxyethyl)-amino)propyl)-1,3-benzodioxole-2,3-dicarboxylate” [Supplementary Concept])) OR (“dorsilurin F” [Supplementary Concept])) OR (“emeriamine” [Supplementary Concept])) OR (“empagliflozin” [Supplementary Concept])) OR (“englitazone” [Supplementary Concept])) OR (“epigallocatechin-3-O-gallate epigallocatechin dimer” [Supplementary Concept])) OR (“ethyl 2-(5-(4-chlorophenyl)pentyl)oxiran-2-carboxylate” [Supplementary Concept])) OR (“etomoxir” [Supplementary Concept])) OR (“Exenatide”[Mesh])) OR (“fenugreek seed meal” [Supplementary Concept])) OR (“firuglipel” [Supplementary Concept])) OR (“ginnalin A” [Supplementary Concept])) OR (“glibornuride” [Supplementary Concept])) OR (“Gliclazide”[Mesh])) OR (“glimepiride” [Supplementary Concept])) OR (“Glipizide”[Mesh])) OR (“gliquidone” [Supplementary Concept])) OR (“glucuronyl glucosamine glycan sulfate” [Supplementary Concept])) OR (“Glyburide”[Mesh])) OR (“gusperimus” [Supplementary Concept])) OR (“inositol phosphate glycan” [Supplementary Concept])) OR (“Insulin”[Mesh])) OR (“ipragliflozin” [Supplementary Concept])) OR (“Islet Amyloid Polypeptide”[Mesh])) OR (“iso-oenothein C” [Supplementary Concept]))) OR (“Hypoglycemic Agents”[Mesh] OR “Hypoglycemic Agents” [Pharmacological Action]))) AND (((“Depression”[Mesh] OR “Depressive Disorder”[Mesh] OR “Depression, Postpartum”[Mesh] OR “Depressive Disorder, Treatment-Resistant”[Mesh] OR “Depressive Disorder, Major”[Mesh] OR “Bipolar Disorder”[Mesh] OR “Affective Disorders, Psychotic”[Mesh] OR “Major Depressive Disorder 1” [Supplementary Concept] OR “Major Depressive Disorder 2” [Supplementary Concept]) OR “Mood Disorders”[Mesh]) OR (((Melancholia*[Title/Abstract]) OR (Depress*[Title/Abstract])) OR (Dysthymi*[Title/Abstract])))) AND (((((((((((Random* adj3 control*[Title/Abstract]) OR (placebo[Title/Abstract])) OR (“Double Blind”[Title/Abstract])) OR (Double-Blind[Title/Abstract])) OR (“Double Masked”[Title/Abstract])) OR (Double-Masked[Title/Abstract])) OR (Single-Blind[Title/Abstract])) OR (Single-Masked[Title/Abstract])) OR (“Single Blind”[Title/Abstract])) OR (“Single Masked”[Title/Abstract])) OR (((“Double-Blind Method”[Mesh]) OR “Single-Blind Method”[Mesh]) OR “Randomized Controlled Trial” [Publication Type]))	
#9	#7 OR #8 Search: ((((((((((Random* adj3 control*[Title/Abstract]) OR (placebo[Title/Abstract])) OR (“Double Blind”[Title/Abstract])) OR (Double-Blind[Title/Abstract])) OR (“Double Masked”[Title/Abstract])) OR (Double-Masked[Title/Abstract])) OR (Single-Blind[Title/Abstract])) OR (Single-Masked[Title/Abstract])) OR (“Single Blind”[Title/Abstract])) OR (“Single Masked”[Title/Abstract])) OR (((“Double-Blind Method”[Mesh]) OR “Single-Blind Method”[Mesh]) OR “Randomized Controlled Trial” [Publication Type])	657,623
#8	Search: (((((((((Random* adj3 control*[Title/Abstract]) OR (placebo[Title/Abstract])) OR (“Double Blind”[Title/Abstract])) OR (Double-Blind[Title/Abstract])) OR (“Double Masked”[Title/Abstract])) OR (Double-Masked[Title/Abstract])) OR (Single-Blind[Title/Abstract])) OR (Single-Masked[Title/Abstract])) OR (“Single Blind”[Title/Abstract])) OR (“Single Masked”[Title/Abstract])	275,470
#7	Search: ((“Double-Blind Method”[Mesh]) OR “Single-Blind Method”[Mesh]) OR “Randomized Controlled Trial” [Publication Type] Sort by: Most Recent	551,997
#6	#4 OR #5 Search: ((“Depression”[Mesh] OR “Depressive Disorder”[Mesh] OR “Depression, Postpartum”[Mesh] OR “Depressive Disorder, Treatment-Resistant”[Mesh] OR “Depressive Disorder, Major”[Mesh] OR “Bipolar Disorder”[Mesh] OR “Affective Disorders, Psychotic”[Mesh] OR “Major Depressive Disorder 1” [Supplementary Concept] OR “Major Depressive Disorder 2” [Supplementary Concept]) OR “Mood Disorders”[Mesh]) OR (((Melancholia*[Title/Abstract]) OR (Depress*[Title/Abstract])) OR (Dysthymi*[Title/Abstract]))	535,971
#5	Search: ((Melancholia*[Title/Abstract]) OR (Depress*[Title/Abstract])) OR (Dysthymi*[Title/Abstract])	465,868
#4	Search: (“Depression”[Mesh] OR “Depressive Disorder”[Mesh] OR “Depression, Postpartum”[Mesh] OR “Depressive Disorder, Treatment-Resistant”[Mesh] OR “Depressive Disorder, Major”[Mesh] OR “Bipolar Disorder”[Mesh] OR “Affective Disorders, Psychotic”[Mesh] OR “Major Depressive Disorder 1” [Supplementary Concept] OR “Major Depressive Disorder 2” [Supplementary Concept]) OR “Mood Disorders”[Mesh] Sort by: Most Recent	257,195
#3	#1 OR #2	489,692
#2	Search: Hypoglycemic*[Title/Abstract] OR Antidiabetic*[Title/Abstract] OR Acarbose[Title/Abstract] OR acarviosine-glucose[Title/Abstract] OR Acetohexamide[Title/Abstract] OR Allicin[Title/Abstract] OR alliin[Title/Abstract] OR alogliptin[Title/Abstract] OR anagliptin[Title/Abstract] OR bexagliflozin[Title/Abstract] OR Biguanides[Title/Abstract] OR Biphasic Insulins[Title/Abstract] OR BRL 26830A[Title/Abstract] OR Buformin[Title/Abstract] OR Butoxamine[Title/Abstract] OR butyl isobutyl phthalate[Title/Abstract] OR Canagliflozin[Title/Abstract] OR Carbutamide[Title/Abstract] OR castanospermine[Title/Abstract] OR Chlorpropamide[Title/Abstract] OR ciglitazone[Title/Abstract] OR CM-10-18 compound[Title/Abstract] OR conduritol B[Title/Abstract] OR deoxyvalidamine[Title/Abstract] OR dihydroacarbose[Title/Abstract] OR diphenyleneiodonium[Title/Abstract] OR diprotin A[Title/Abstract] OR dorsilurin F[Title/Abstract] OR dulaglutide[Title/Abstract] OR emeriamine[Title/Abstract] OR empagliflozin[Title/Abstract] OR englitazone[Title/Abstract] OR etomoxir[Title/Abstract] OR Exenatide[Title/Abstract] OR fenugreek seed meal[Title/Abstract] OR firuglipel[Title/Abstract] OR ginnalin A[Title/Abstract] OR glibornuride[Title/Abstract] OR Gliclazide[Title/Abstract] OR glimepiride[Title/Abstract] OR Glipizide[Title/Abstract] OR gliquidone[Title/Abstract] OR Glyburide[Title/Abstract] OR Gusperimus[Title/Abstract] OR inositol phosphate glycan[Title/Abstract] OR Insulin[Title/Abstract] OR Ipragliflozin[Title/Abstract] OR Islet Amyloid Polypeptide[Title/Abstract] OR iso-oenothein C[Title/Abstract] OR isoacarbose[Title/Abstract] OR jamutannin A[Title/Abstract] OR jamutannin B[Title/Abstract] OR lamesticumin G[Title/Abstract] OR Linagliptin[Title/Abstract] OR lipoyl vildagliptin[Title/Abstract] OR Liraglutide[Title/Abstract] OR Lixisenatide[Title/Abstract] OR Lonchocarpene[Title/Abstract] OR meglitinide[Title/Abstract] OR Metformin[Title/Abstract] OR midaglizole[Title/Abstract] OR miglitol[Title/Abstract] OR miglustat[Title/Abstract] OR mitiglinide[Title/Abstract] OR MK-2640[Title/Abstract] OR MR1704[Title/Abstract] OR Nateglinide[Title/Abstract] OR nigrosporamide A[Title/Abstract] OR NN 7201[Title/Abstract] OR Peroxovanadate[Title/Abstract] OR Phenformin[Title/Abstract] OR phenyl biguanide[Title/Abstract] OR Pioglitazone[Title/Abstract] OR pistagremic acid[Title/Abstract] OR polyflavanostilbene A[Title/Abstract] OR ponalrestat[Title/Abstract] OR pradimicin Q[Title/Abstract] OR pramlintide[Title/Abstract] OR purunuside A[Title/Abstract] OR purunuside B[Title/Abstract] OR purunuside C[Title/Abstract] OR pycnalin[Title/Abstract] OR remogliflozin etabonate[Title/Abstract] OR repaglinide[Title/Abstract] OR Rosiglitazone[Title/Abstract] OR rubasperone A[Title/Abstract] OR rubasperone B[Title/Abstract] OR S009-0629[Title/Abstract] OR salicortin[Title/Abstract] OR saxagliptin[Title/Abstract] OR schulzeine A[Title/Abstract] OR scirpusin C[Title/Abstract] OR sergliflozin etabonate[Title/Abstract] OR Sitagliptin Phosphate[Title/Abstract] OR Sulphostin[Title/Abstract] OR Syrrx 106124[Title/Abstract] OR tirzepatide[Title/Abstract] OR Tolazamide[Title/Abstract] OR Tolbutamide[Title/Abstract] OR Topiramate[Title/Abstract] OR Trestatin[Title/Abstract] OR Troglitazone[Title/Abstract] OR valienamine[Title/Abstract] OR vanadyl sulfate[Title/Abstract] OR velagliflozin[Title/Abstract] OR Vildagliptin[Title/Abstract] OR Voglibose[Title/Abstract] OR Volagidemab[Title/Abstract] OR Xultophy[Title/Abstract] OR zopolrestat[Title/Abstract]	422,360
#1	Search: (((((((((((((((((((((((((((((((((((((((((((((((((((((((((((((((((((((“zopolrestat” [Supplementary Concept]) OR (“Xultophy” [Supplementary Concept])) OR (“volagidemab” [Supplementary Concept])) OR (“voglibose” [Supplementary Concept])) OR (“Vildagliptin”[Mesh])) OR (“velagliflozin” [Supplementary Concept])) OR (“vanadyl sulfate” [Supplementary Concept])) OR (“valienamine” [Supplementary Concept])) OR (“Troglitazone”[Mesh])) OR (“trestatin” [Supplementary Concept])) OR (“Topiramate”[Mesh])) OR (“Tolbutamide”[Mesh])) OR (“Tolazamide”[Mesh])) OR (“TMC 2C” [Supplementary Concept])) OR (“TMC 2B” [Supplementary Concept])) OR (“tirzepatide” [Supplementary Concept])) OR (“Syrrx 106124” [Supplementary Concept])) OR (“sulphostin” [Supplementary Concept])) OR (“Sitagliptin Phosphate”[Mesh])) OR (“sergliflozin etabonate” [Supplementary Concept])) OR (“schulzeine A” [Supplementary Concept])) OR (“saxagliptin” [Supplementary Concept])) OR (“salicortin” [Supplementary Concept])) OR (“S009-0629” [Supplementary Concept])) OR (“rubasperone B” [Supplementary Concept])) OR (“rubasperone A” [Supplementary Concept])) OR (“Rosiglitazone”[Mesh])) OR (“repaglinide” [Supplementary Concept])) OR (“remogliflozin etabonate” [Supplementary Concept])) OR (“pycnalin” [Supplementary Concept])) OR (“purunuside C” [Supplementary Concept])) OR (“purunuside B” [Supplementary Concept])) OR (“purunuside A” [Supplementary Concept])) OR (“pramlintide” [Supplementary Concept])) OR (“pradimicin Q” [Supplementary Concept])) OR (“ponalrestat” [Supplementary Concept])) OR (“polyflavanostilbene A” [Supplementary Concept])) OR (“pistagremic acid” [Supplementary Concept])) OR (“Pioglitazone”[Mesh])) OR (“phenyl-6-deoxy-6-(morpholin-4-yl)glucopyranoside” [Supplementary Concept])) OR (“phenyl biguanide” [Supplementary Concept])) OR (“Phenformin”[Mesh])) OR (“peroxovanadate” [Supplementary Concept])) OR (“NN 7201” [Supplementary Concept])) OR (“nigrosporamide A” [Supplementary Concept])) OR (“Nateglinide”[Mesh])) OR (“*N*-phenyl-4,5,6,7-tetrachlorophthalimide” [Supplementary Concept])) OR (“*N*-nonyl-1-deoxynojirimycin” [Supplementary Concept])) OR (“*N*-alanylproline-O-(4-nitrobenzoyl)hydroxylamine” [Supplementary Concept])) OR (“MYL-1501D” [Supplementary Concept])) OR (“MR1704” [Supplementary Concept])) OR (“MK-2640” [Supplementary Concept])) OR (“mitiglinide” [Supplementary Concept])) OR (“miglustat” [Supplementary Concept])) OR (“miglitol” [Supplementary Concept])) OR (“midaglizole” [Supplementary Concept])) OR (“methyl 2-tetradecylglycidate” [Supplementary Concept])) OR (“methionyl-2-cyanopyrrolidine” [Supplementary Concept])) OR (“meglitinide” [Supplementary Concept])) OR (“lonchocarpene” [Supplementary Concept])) OR (“lixisenatide” [Supplementary Concept])) OR (“Liraglutide”[Mesh])) OR (“lipoyl vildagliptin” [Supplementary Concept])) OR (“Linagliptin”[Mesh])) OR (“lamesticumin G” [Supplementary Concept])) OR (“jamutannin B” [Supplementary Concept])) OR (“jamutannin A” [Supplementary Concept])) OR (“isoacarbose” [Supplementary Concept])) OR ((((((((((((((((((((((((((((((((((((((((((((((((((((((((((((((((((((((((((((((((((((((((“(2S,3R,4R,5S,6R)-2-(4-chloro-3-(4-ethoxybenzyl)phenyl)-6-(methylthio)tetrahydro-2H-pyran-3,4,5-triol” [Supplementary Concept]) OR (“(3,3-difluoropyrrolidin-1-yl)-(4-(4-pyrimidin-2-yl-piperazin-1-yl)-pyrrolidin-2-yl)methanone” [Supplementary Concept])) OR (“1,4-dideoxy-1,4-((2,3,4,5,6-pentahydroxyhexyl)episulfoniumylidene)arabinitol” [Supplementary Concept])) OR (“1-(((1-(hydroxymethyl)cyclopentyl)amino)acetyl)pyrrolidine-2,5-dicarbonitrile” [Supplementary Concept])) OR (“1-(((2-((5-cyanopyridin-2-yl)amino)ethyl)amino)acetyl)-2-cyano-(S)-pyrrolidine” [Supplementary Concept])) OR (“1-((6-hydroxy-2-azabicyclo(2.2.1)hept-3-yl)carbonyl)-2-pyrrolidinecarbonitrile” [Supplementary Concept])) OR (“1-(2’-amino-3’,3’-dimethylbutanoyl)pyrrolidine-2-carbonitrile” [Supplementary Concept])) OR (“1-(2-(5-cyano-2-pyridinylamino)ethylamino)acetyl-2-pyrrolidinecarbonitrile” [Supplementary Concept])) OR (“1-(4-aminomethyl-4-(3-chlorophenyl)cyclohexyl)-tetrahydropyrimidin-2-one” [Supplementary Concept])) OR (“13-hydroxykompasinol A” [Supplementary Concept])) OR (“2,3,4,5,6-pentagalloylglucose” [Supplementary Concept])) OR (“2,4-thiazolidinedione” [Supplementary Concept])) OR (“2-(3-(4-ethoxybenzyl)-4-chlorophenyl)-6-hydroxymethyltetrahydro-2H-pyran-3,4,5-triol” [Supplementary Concept])) OR (“2-bromopalmitate” [Supplementary Concept])) OR (“2-tetradecylglycidic acid” [Supplementary Concept])) OR (“24,25,26,27-tetranor-apotirucalla-(apoeupha)-1-senecioyloxy-3,7-dihydroxy-14,20,22-trien-21,23-epoxy” [Supplementary Concept])) OR (“3,5-dimethoxy-4’-O-prenyl-trans-stilbene” [Supplementary Concept])) OR (“3-amino-1-(1-boronic-ethyl)pyrrolidine-2-one” [Supplementary Concept])) OR (“3-amino-4-(2,4,5-trifluorophenyl)-*N*-(4-(6-(2-methoxyethoxy)benzothiazol-2-yl)tetrahydropyran-4-yl)butanamide” [Supplementary Concept])) OR (“3-amino-4-(3,3-difluoropyrrolidin-1-yl)-*N*,*N*-dimethyl-4-oxo-2-(4-(1,2,4)triazolo(1,5-a)pyridin-6-ylphenyl)butanamide” [Supplementary Concept])) OR (“3-O-digalloyl-1,2,4,6-tetra-O-galloylglucose” [Supplementary Concept])) OR (“4-(3-amino-4-(2,4,5-trifluorophenyl)butanoyl)-3-(2,2,2-trifluoroethyl)-1,4-diazepan-2-one” [Supplementary Concept])) OR (“4-(8-(4-fluorobenzyl)-3-(trifluoromethyl)-5,6-dihydro(1,2,4)triazolo(4,3-a)pyrazin-7(8H)-yl)-4-oxo-1-(2,4,5-trifluorophenyl)butan-2-amine” [Supplementary Concept])) OR (“4-(8-(4-fluorobenzyl)-3-(trifluoromethyl)-5,6-dihydro(1,2,4)triazolo(4,3-a)pyrazin-7(8H)-yl)-4-oxo-1-(2,4,5-trifluorophenyl)butan-2-amine” [Supplementary Concept] AND “4-(8-methyl-3-(trifluoromethyl)-5,6-dihydro(1,2,4)triazolo(4,3-a)pyrazin-7(8H)-yl)-4-oxo-1-(2,4,5-trifluorophenyl)butan-2-amine” [Supplementary Concept])) OR (“4-O-glucopyranosylmoranoline” [Supplementary Concept])) OR (“5,7-dihydroxy-6,8-dimethyl-4’-methoxyflavone” [Supplementary Concept])) OR (“5-amino-5-deoxygluconic acid delta-lactam” [Supplementary Concept])) OR (“6-((4-ethylphenyl)methyl)-3’,4’,5’,6’-tetrahydro-6’-(hydroxymethyl)spiro(isobenzofuran-1(3H),2’-(2H)pyran)-3’,4’,5’-triol” [Supplementary Concept])) OR (“6-amino-pyrido(2,3-d)pyrimidine-2,4-dione” [Supplementary Concept])) OR (“6-aminocyclohex-4-ene-1,2,3-triol” [Supplementary Concept])) OR (“6-methyl-3-hydroxypiperidine-2-carboxylic acid” [Supplementary Concept])) OR (“7-deoxycastanospermine” [Supplementary Concept])) OR (“8-(2-hydroxypropan-2-yl)-5-hydroxy-7-methoxy-6-methyl-4’-methoxyflavone” [Supplementary Concept])) OR (“8-hydroxy-2,2,14,14-tetramethylpentadecanedioic acid” [Supplementary Concept])) OR (“ABT 341” [Supplementary Concept])) OR (“Acarbose”[Mesh])) OR (“acarviosinyl-(1-9)-3-glucopyranosylpropene” [Supplementary Concept])) OR (“Acetohexamide”[Mesh])) OR (“AICA ribonucleotide” [Supplementary Concept])) OR (“allicin” [Supplementary Concept])) OR (“alliin” [Supplementary Concept])) OR (“alogliptin” [Supplementary Concept])) OR (“anagliptin” [Supplementary Concept])) OR (“bexagliflozin” [Supplementary Concept])) OR (“Biguanides”[Mesh])) OR (“Biphasic Insulins”[Mesh])) OR (“BRL 26830A” [Supplementary Concept])) OR (“Buformin”[Mesh])) OR (“Butoxamine”[Mesh])) OR (“butyl isobutyl phthalate” [Supplementary Concept])) OR (“Canagliflozin”[Mesh])) OR (“Carbutamide”[Mesh])) OR (“castanospermine” [Supplementary Concept])) OR (“Chlorpropamide”[Mesh])) OR (“ciglitazone” [Supplementary Concept])) OR (“CM-10-18 compound” [Supplementary Concept])) OR (“conduritol B” [Supplementary Concept])) OR (“copper bis(3,5-diisopropylsalicylate)” [Supplementary Concept])) OR (“deoxyvalidamine” [Supplementary Concept])) OR (“dihydroacarbose” [Supplementary Concept])) OR (“dihydropyrano(3,2-c)quinoline” [Supplementary Concept])) OR (“diphenyleneiodonium” [Supplementary Concept])) OR (“diprotin A” [Supplementary Concept])) OR (“disodium (R,R)-5-(2-((2-(3-chlorophenyl)-2-hydroxyethyl)-amino)propyl)-1,3-benzodioxole-2,3-dicarboxylate” [Supplementary Concept])) OR (“dorsilurin F” [Supplementary Concept])) OR (“emeriamine” [Supplementary Concept])) OR (“empagliflozin” [Supplementary Concept])) OR (“englitazone” [Supplementary Concept])) OR (“epigallocatechin-3-O-gallate epigallocatechin dimer” [Supplementary Concept])) OR (“ethyl 2-(5-(4-chlorophenyl)pentyl)oxiran-2-carboxylate” [Supplementary Concept])) OR (“etomoxir” [Supplementary Concept])) OR (“Exenatide”[Mesh])) OR (“fenugreek seed meal” [Supplementary Concept])) OR (“firuglipel” [Supplementary Concept])) OR (“ginnalin A” [Supplementary Concept])) OR (“glibornuride” [Supplementary Concept])) OR (“Gliclazide”[Mesh])) OR (“glimepiride” [Supplementary Concept])) OR (“Glipizide”[Mesh])) OR (“gliquidone” [Supplementary Concept])) OR (“glucuronyl glucosamine glycan sulfate” [Supplementary Concept])) OR (“Glyburide”[Mesh])) OR (“gusperimus” [Supplementary Concept])) OR (“inositol phosphate glycan” [Supplementary Concept])) OR (“Insulin”[Mesh])) OR (“ipragliflozin” [Supplementary Concept])) OR (“Islet Amyloid Polypeptide”[Mesh])) OR (“iso-oenothein C” [Supplementary Concept]))) OR (“Hypoglycemic Agents”[Mesh] OR “Hypoglycemic Agents” [Pharmacological Action])	268,317
